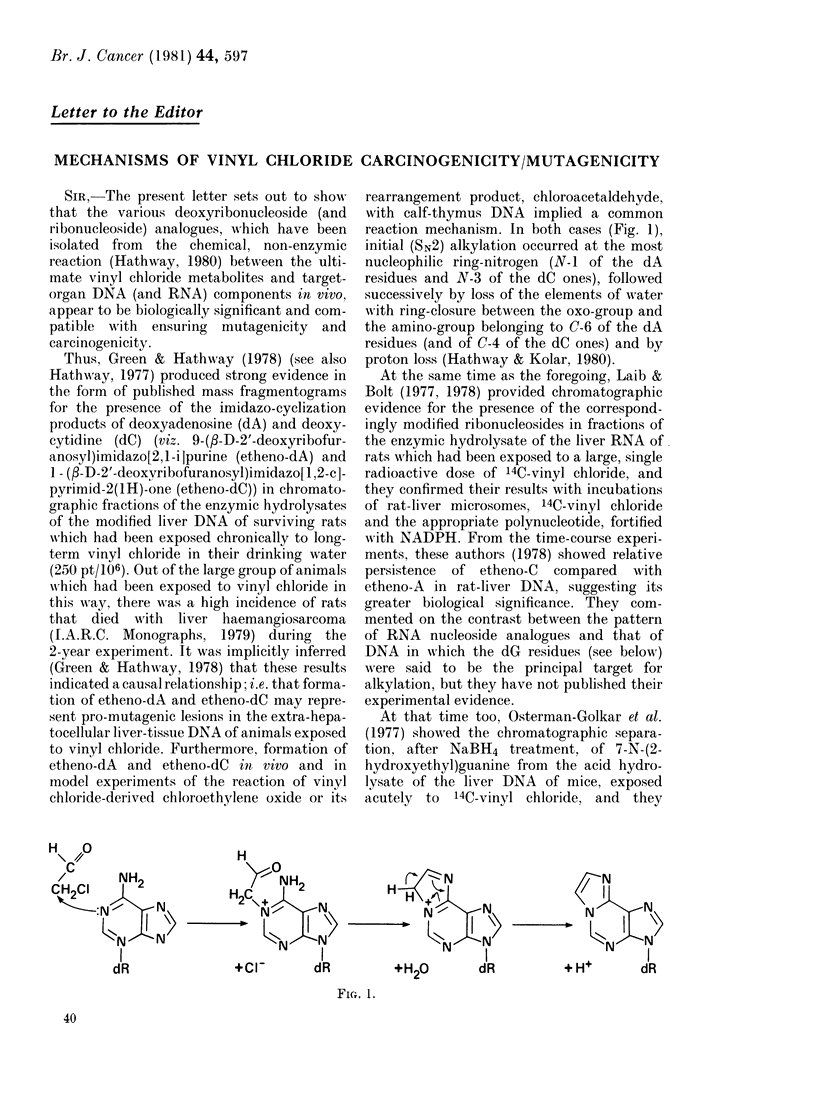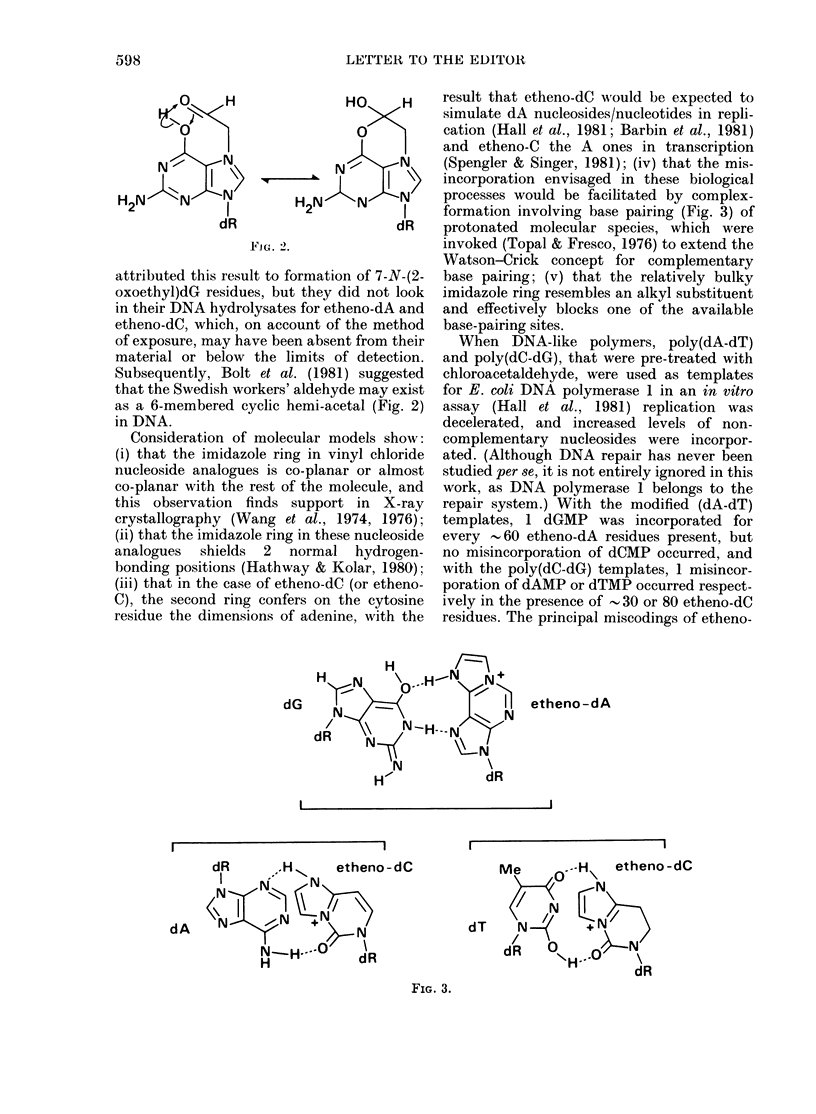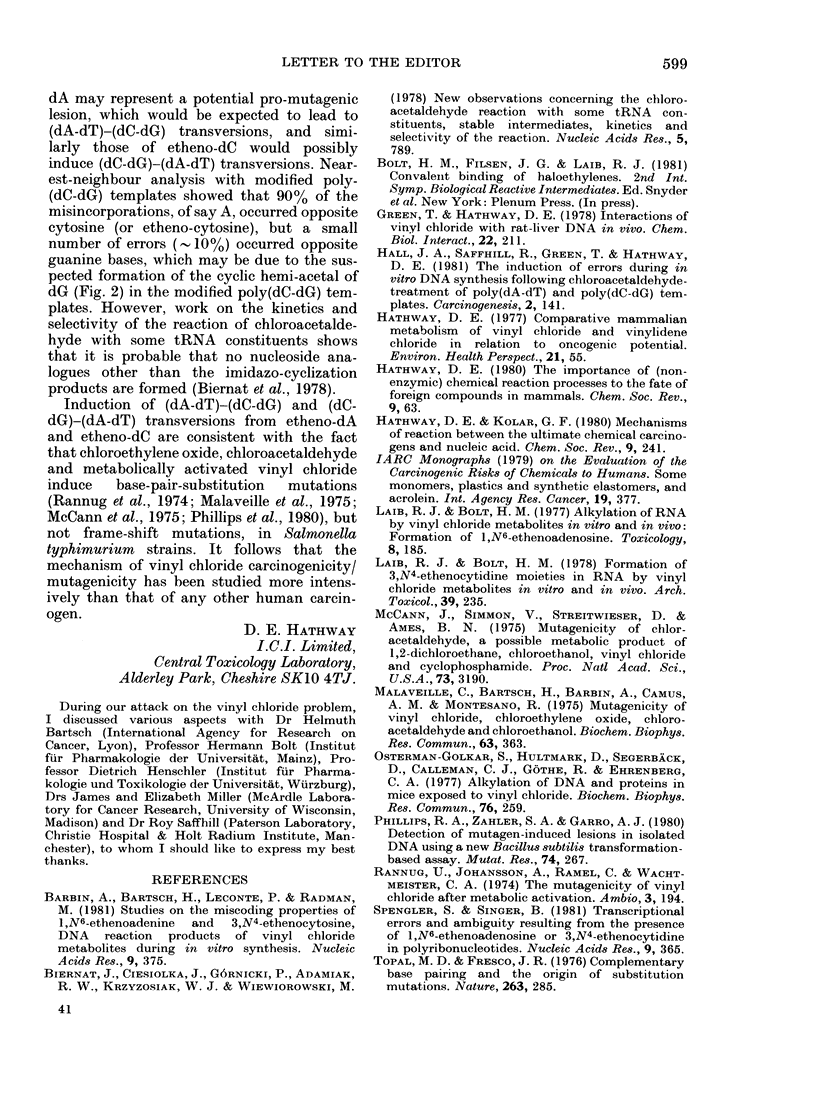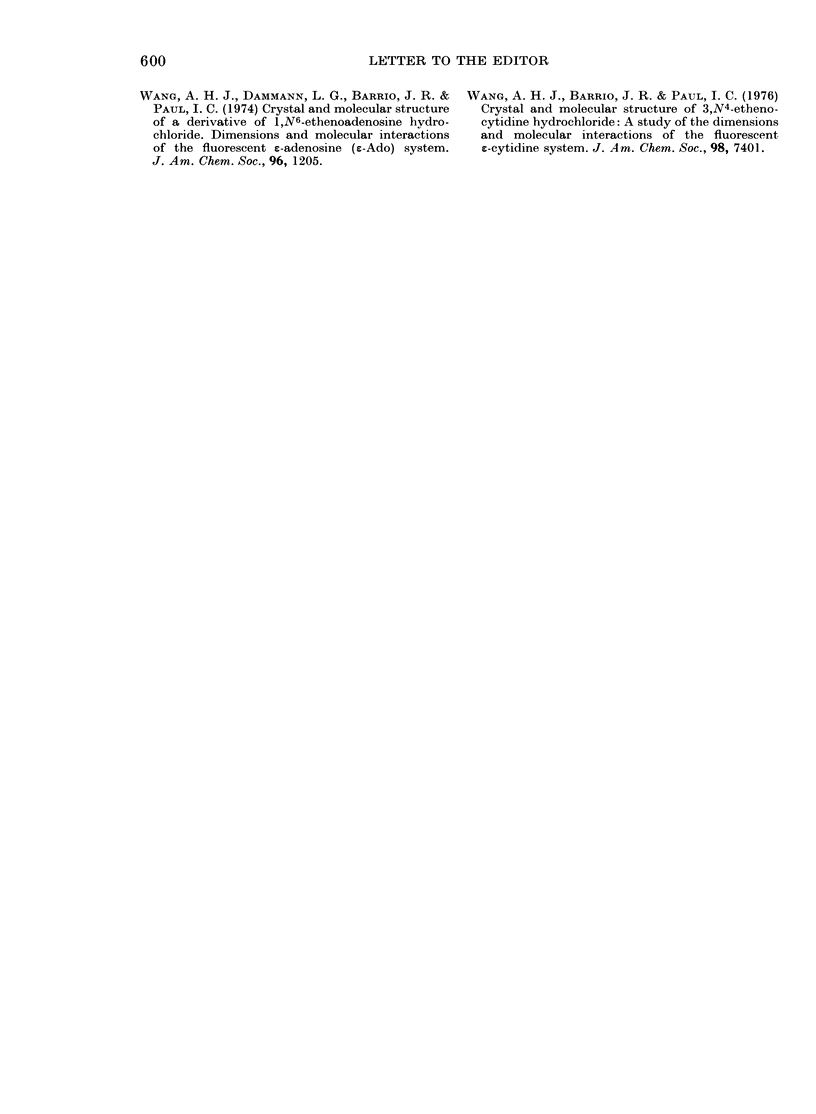# Mechanisms of vinyl chloride carcinogenicity/mutagenicity.

**DOI:** 10.1038/bjc.1981.233

**Published:** 1981-10

**Authors:** D. E. Hathaway


*Br. J. Cancer* (1981) **44**, 597

***Letter to the Editor***

### MECHANISMS OF VINYL CHLORIDE CARCINOGENICITY/MUTAGENICITY

SIR,—The present letter sets out to show that the various deoxyribonucleoside (and ribonucleoside) analogues, which have been isolated from the chemical, non-enzymic reaction (Hathway, 1980) between the ultimate vinyl chloride metabolites and target-organ DNA (and RNA) components *in vivo*, appear to be biologically significant and compatible with ensuring mutagenicity and carcinogenicity.

Thus, Green & Hathway (1978) (see also Hathway, 1977) produced strong evidence in the form of published mass fragmentograms for the presence of the imidazo-cyclization products of deoxyadenosine (dA) and deoxycytidine (dC) (*viz.* 9-(β-D-2′-deoxyribofuranosyl)imidazo[2,1-i]purine (etheno-dA) and 1-(β-D-2′-deoxyribofuranosyl)imidazo[1,2-c]-pyrimid-2(1H)-one (etheno-dC)) in chromatographic fractions of the enzymic hydrolysates of the modified liver DNA of surviving rats which had been exposed chronically to long-term vinyl chloride in their drinking water (250 pt/$10^6$). Out of the large group of animals which had been exposed to vinyl chloride in this way, there was a high incidence of rats that died with liver haemangiosarcoma (I.A.R.C. Monographs, 1979) during the 2-year experiment. It was implicitly inferred (Green & Hathway, 1978) that these results indicated a causal relationship; *i.e.* that formation of etheno-dA and etheno-dC may represent pro-mutagenic lesions in the extra-hepatocellular liver-tissue DNA of animals exposed to vinyl chloride. Furthermore, formation of etheno-dA and etheno-dC *in vivo* and in model experiments of the reaction of vinyl chloride-derived chloroethylene oxide or its rearrangement product, chloroacetaldehyde, with calf-thymus DNA implied a common reaction mechanism. In both cases (Fig. 1), initial ($S_N2$) alkylation occurred at the most nucleophilic ring-nitrogen (*N*-1 of the dA residues and *N*-3 of the dC ones), followed successively by loss of the elements of water with ring-closure between the oxo-group and the amino-group belonging to *C*-6 of the dA residues (and of *C*-4 of the dC ones) and by proton loss (Hathway & Kolar, 1980).

At the same time as the foregoing, Laib & Bolt (1977, 1978) provided chromatographic evidence for the presence of the correspondingly modified ribonucleosides in fractions of the enzymic hydrolysate of the liver RNA of rats which had been exposed to a large, single radioactive dose of $^{14}C$-vinyl chloride, and they confirmed their results with incubations of rat-liver microsomes, $^{14}C$-vinyl chloride and the appropriate polynucleotide, fortified with NADPH. From the time-course experiments, these authors (1978) showed relative persistence of etheno-C compared with etheno-A in rat-liver DNA, suggesting its greater biological significance. They commented on the contrast between the pattern of RNA nucleoside analogues and that of DNA in which the dG residues (see below) were said to be the principal target for alkylation, but they have not published their experimental evidence.

At that time too, Osterman-Golkar *et al.* (1977) showed the chromatographic separation, after $NaBH_4$ treatment, of 7-N-(2-hydroxyethyl)guanine from the acid hydrolysate of the liver DNA of mice, exposed acutely to $^{14}C$-vinyl chloride, and they

FIG. 1.

FIG. 2.

attributed this result to formation of 7-*N*-(2-oxoethyl)dG residues, but they did not look in their DNA hydrolysates for etheno-dA and etheno-dC, which, on account of the method of exposure, may have been absent from their material or below the limits of detection. Subsequently, Bolt *et al.* (1981) suggested that the Swedish workers' aldehyde may exist as a 6-membered cyclic hemi-acetal (Fig. 2) in DNA.

Consideration of molecular models show: (i) that the imidazole ring in vinyl chloride nucleoside analogues is co-planar or almost co-planar with the rest of the molecule, and this observation finds support in X-ray crystallography (Wang *et al.*, 1974, 1976); (ii) that the imidazole ring in these nucleoside analogues shields 2 normal hydrogen-bonding positions (Hathway & Kolar, 1980); (iii) that in the case of etheno-dC (or etheno-C), the second ring confers on the cytosine residue the dimensions of adenine, with the result that etheno-dC would be expected to simulate dA nucleosides/nucleotides in replication (Hall *et al.*, 1981; Barbin *et al.*, 1981) and etheno-C the A ones in transcription (Spengler & Singer, 1981); (iv) that the misincorporation envisaged in these biological processes would be facilitated by complex-formation involving base pairing (Fig. 3) of protonated molecular species, which were invoked (Topal & Fresco, 1976) to extend the Watson–Crick concept for complementary base pairing; (v) that the relatively bulky imidazole ring resembles an alkyl substituent and effectively blocks one of the available base-pairing sites.

When DNA-like polymers, poly(dA-dT) and poly(dC-dG), that were pre-treated with chloroacetaldehyde, were used as templates for *E. coli* DNA polymerase 1 in an *in vitro* assay (Hall *et al.*, 1981) replication was decelerated, and increased levels of non-complementary nucleosides were incorporated. (Although DNA repair has never been studied *per se*, it is not entirely ignored in this work, as DNA polymerase 1 belongs to the repair system.) With the modified (dA-dT) templates, 1 dGMP was incorporated for every ~60 etheno-dA residues present, but no misincorporation of dCMP occurred, and with the poly(dC-dG) templates, 1 misincorporation of dAMP or dTMP occurred respectively in the presence of ~30 or 80 etheno-dC residues. The principal miscodings of etheno-

FIG. 3.

dA may represent a potential pro-mutagenic lesion, which would be expected to lead to (dA-dT)-(dC-dG) transversions, and similarly those of etheno-dC would possibly induce (dC-dG)-(dA-dT) transversions. Nearest-neighbour analysis with modified poly-(dC-dG) templates showed that 90% of the misincorporations, of say A, occurred opposite cytosine (or etheno-cytosine), but a small number of errors (~10%) occurred opposite guanine bases, which may be due to the suspected formation of the cyclic hemi-acetal of dG (Fig. 2) in the modified poly(dC-dG) templates. However, work on the kinetics and selectivity of the reaction of chloroacetaldehyde with some tRNA constituents shows that it is probable that no nucleoside analogues other than the imidazo-cyclization products are formed (Biernat *et al.*, 1978).

Induction of (dA-dT)-(dC-dG) and (dC-dG)-(dA-dT) transversions from etheno-dA and etheno-dC are consistent with the fact that chloroethylene oxide, chloroacetaldehyde and metabolically activated vinyl chloride induce base-pair-substitution mutations (Rannug *et al.*, 1974; Malaveille *et al.*, 1975; McCann *et al.*, 1975; Phillips *et al.*, 1980), but not frame-shift mutations, in *Salmonella typhimurium* strains. It follows that the mechanism of vinyl chloride carcinogenicity/mutagenicity has been studied more intensively than that of any other human carcinogen.

D. E. HATHWAY
*I.C.I. Limited,*
*Central Toxicology Laboratory,*
*Alderley Park, Cheshire SK10 4TJ.*

During our attack on the vinyl chloride problem, I discussed various aspects with Dr Helmuth Bartsch (International Agency for Research on Cancer, Lyon), Professor Hermann Bolt (Institut für Pharmakologie der Universität, Mainz), Professor Dietrich Henschler (Institut für Pharmakologie und Toxikologie der Universität, Würzburg), Drs James and Elizabeth Miller (McArdle Laboratory for Cancer Research, University of Wisconsin, Madison) and Dr Roy Saffhill (Paterson Laboratory, Christie Hospital & Holt Radium Institute, Manchester), to whom I should like to express my best thanks.

### REFERENCES

BARBIN, A., BARTSCH, H., LECONTE, P. & RADMAN, M. (1981) Studies on the miscoding properties of 1,$N^6$-ethenoadenine and 3,$N^4$-ethenocytosine, DNA reaction products of vinyl chloride metabolites during *in vitro* synthesis. *Nucleic Acids Res.*, **9**, 375.

BIERNAT, J., CIESIOLKA, J., GÓRNICKI, P., ADAMIAK, R. W., KRZYZOSIAK, W. J. & WIEWIOROWSKI, M. (1978) New observations concerning the chloroacetaldehyde reaction with some tRNA constituents, stable intermediates, kinetics and selectivity of the reaction. *Nucleic Acids Res.*, **5**, 789.

BOLT, H. M., FILSEN, J. G. & LAIB, R. J. (1981) Convalent binding of haloethylenes. *2nd Int. Symp. Biological Reactive Intermediates.* Ed. Snyder *et al.* New York: Plenum Press. (In press).

GREEN, T. & HATHWAY, D. E. (1978) Interactions of vinyl chloride with rat-liver DNA *in vivo. Chem. Biol. Interact.*, **22**, 211.

HALL, J. A., SAFFHILL, R., GREEN, T. & HATHWAY, D. E. (1981) The induction of errors during *in vitro* DNA synthesis following chloroacetaldehyde-treatment of poly(dA-dT) and poly(dC-dG) templates. *Carcinogenesis*, **2**, 141.

HATHWAY, D. E. (1977) Comparative mammalian metabolism of vinyl chloride and vinylidene chloride in relation to oncogenic potential. *Environ. Health Perspect.*, **21**, 55.

HATHWAY, D. E. (1980) The importance of (non-enzymic) chemical reaction processes to the fate of foreign compounds in mammals. *Chem. Soc. Rev.*, **9**, 63.

HATHWAY, D. E. & KOLAR, G. F. (1980) Mechanisms of reaction between the ultimate chemical carcinogens and nucleic acid. *Chem. Soc. Rev.*, **9**, 241.

*IARC Monographs* (1979) *on the Evaluation of the Carcinogenic Risks of Chemicals to Humans.* Some monomers, plastics and synthetic elastomers, and acrolein. *Int. Agency Res. Cancer*, **19**, 377.

LAIB, R. J. & BOLT, H. M. (1977) Alkylation of RNA by vinyl chloride metabolites *in vitro* and *in vivo*: Formation of 1,$N^6$-ethenoadenosine. *Toxicology*, **8**, 185.

LAIB, R. J. & BOLT, H. M. (1978) Formation of 3,$N^4$-ethenocytidine moieties in RNA by vinyl chloride metabolites *in vitro* and *in vivo. Arch. Toxicol.*, **39**, 235.

MCCANN, J., SIMMON, V., STREITWIESER, D. & AMES, B. N. (1975) Mutagenicity of chloracetaldehyde, a possible metabolic product of 1,2-dichloroethane, chloroethanol, vinyl chloride and cyclophosphamide. *Proc. Natl Acad. Sci., U.S.A.*, **73**, 3190.

MALAVEILLE, C., BARTSCH, H., BARBIN, A., CAMUS, A. M. & MONTESANO, R. (1975) Mutagenicity of vinyl chloride, chloroethylene oxide, chloroacetaldehyde and chloroethanol. *Biochem. Biophys. Res. Commun.*, **63**, 363.

OSTERMAN-GOLKAR, S., HULTMARK, D., SEGERBÄCK, D., CALLEMAN, C. J., GÖTHE, R. & EHRENBERG, C. A. (1977) Alkylation of DNA and proteins in mice exposed to vinyl chloride. *Biochem. Biophys. Res. Commun.*, **76**, 259.

PHILLIPS, R. A., ZAHLER, S. A. & GARRO, A. J. (1980) Detection of mutagen-induced lesions in isolated DNA using a new *Bacillus subtilis* transformation-based assay. *Mutat. Res.*, **74**, 267.

RANNUG, U., JOHANSSON, A., RAMEL, C. & WACHTMEISTER, C. A. (1974) The mutagenicity of vinyl chloride after metabolic activation. *Ambio*, **3**, 194.

SPENGLER, S. & SINGER, B. (1981) Transcriptional errors and ambiguity resulting from the presence of 1,$N^6$-ethenoadenosine or 3,$N^4$-ethenocytidine in polyribonucleotides. *Nucleic Acids Res.*, **9**, 365.

TOPAL, M. D. & FRESCO, J. R. (1976) Complementary base pairing and the origin of substitution mutations. *Nature*, **263**, 285.

Wang, A. H. J., Dammann, L. G., Barrio, J. R. & Paul, I. C. (1974) Crystal and molecular structure of a derivative of 1,$N^6$-ethenoadenosine hydrochloride. Dimensions and molecular interactions of the fluorescent ε-adenosine (ε-Ado) system. *J. Am. Chem. Soc.*, **96,** 1205.

Wang, A. H. J., Barrio, J. R. & Paul, I. C. (1976) Crystal and molecular structure of 3,$N^4$-ethenocytidine hydrochloride: A study of the dimensions and molecular interactions of the fluorescent ε-cytidine system. *J. Am. Chem. Soc.*, **98,** 7401.